# The Real Experience of Lay Responders Performing Cardiopulmonary Resuscitation: A Synthesis of Qualitative Evidence

**DOI:** 10.3389/phrs.2024.1606650

**Published:** 2024-06-05

**Authors:** Na Li, Chen Shen, Xin Yang, Rao Wang, Lian Qi Gu, Wei Zhao, Zhi Ping Chu

**Affiliations:** ^1^ Second Affiliated Hospital, Nanjing Medical University, Nanjing, Jiangsu, China; ^2^ Nanjing Medical University, Nanjing, Jiangsu, China; ^3^ Shanghai Mental Health Center, School of Medicine, Shanghai Jiao Tong University, Shanghai, China

**Keywords:** cardiopulmonary resuscitation, lay responder, real experience, qualitative synthesis, psychological intervention

## Abstract

**Objectives:**

To synthesize qualitative evidence on the experience of lay responders performing cardiopulmonary resuscitation (CPR).

**Methods:**

Qualitative evidence synthesis was performed using the Thomas and Harden method. The PubMed, Cochrane Library, Web of Science, OVID Medline, Embase, CINAHL, CNKI, and WanFang databases were systematically searched. The quality of the research was assessed by the Critical Assessment Skills Program Tool (CASP).

**Results:**

A total of 5,610 studies were identified, and 9 studies were included in the analysis. Four analytical themes were generated: emotional ambivalence before CPR, psychological tolerance during CPR, perceived experience after CPR, and enhancing psychological resilience.

**Conclusion:**

Lay responders face complicated psychological experience during CPR, which may be susceptible to psychological effects such as “loss aversion,” “bystander effects” and “knowledge curse.” In addition to the timely retraining of CPR, lay responders should be instructed to manage psychological distress and improve psychological resilience. More importantly, the psychological sequelae may be long-lasting, requiring ongoing psychological intervention and follow-up based on valuing transdisciplinarity across endeavours.

## Introduction

Out-of-hospital cardiac arrest (OHCA) is a critical public health concern characterized by high mortality and morbidity and has been identified as contributing factors, including congenital heart disease [1], acute coronary syndrome [2], air pollution [3], viral infections [4], drug abuse [5], etc. Given that cardiac arrest often occurs outside of a medical setting, but the golden time for resuscitation is only 4 min, bystander cardiopulmonary resuscitation (CPR) is essential to save patients’ lives [6]. Ample evidence [7–10] suggests that bystander CPR significantly increases patients’ chances of survival, but without timely and effective CPR and defibrillation, survival rates are less than half, and neurological outcomes are worse [11]. Given these concerns, there is wide consensus on disseminating education on bystander CPR among the general public and even incorporating it into the curriculum for school-aged children [12] to rapidly recognize signs of cardiac arrest and perform immediate and effective CPR.

However, it is of note that the percentage of OHCA patients receiving bystander CPR is significantly low worldwide, ranging between 35% and 45% [13], notwithstanding a myriad of CPR-related training for lay responders [14]. Towards this end, extensive public education has been conducted to encourage lay responders to initiate CPR for unconscious victims who are not breathing [15], but this still cannot adequately prepare lay responders for OHCA patients. There is a growing appreciation that psychological factors may play important roles throughout CPR and even affect future psychological and behavioral performance [16, 17], as lay responders have to face great psychological stress and fear during the whole process of CPR [18], which impacts the decision-making and execution of CPR [19]. In addition, caution is needed after CPR due to the reason that lay responders may experience psychological sequelae for a long time (e.g., self-blame for not providing effective CPR, guilt from fear of poor patient prognosis) and even progressively suffer from posttraumatic stress disorder (PTSD) [20].

There is a critical need for lay responders to overcome fear in the actual rescue process [21] and be equipped with courage, self-confidence and positive attitudinal beliefs [14, 22]. In this regard, stress coping strategies [23] or virtual reality-based CPR simulation training [24] are increasingly being used throughout the CPR training process, and these strategies have been proven to be effective in reducing the attitude, intention and perception of stress among lay responders. Despite this, considering the complexity of the psychological experience of CPR, including the duration of CPR, the prognosis of patients and the attitudes of professional rescuers may have important impacts on the psychological experience of lay responders [17]. It is necessary to expand the training of CPR to include the psychological experience of lay responders [25, 26], which may have important reference value for improving the attitudes and willingness of lay responders toward CPR.

To reduce the incidence of psychological sequelae, improve self-perception of the implementation of CPR and increase the response rate, qualitative studies [26–28] have been conducted to explore the feelings, experience, and coping strategies of lay responders performing CPR in recent years. By gaining a deeper understanding of the challenges and experience faced by lay responders, healthcare providers can better equip them with the mental health support and training needed to provide effective CPR in OHCA patients, save lives and reduce the risk of brain injury. However, individual primary studies involve different cultural backgrounds, methodologies, and values, which may not comprehensively reflect the psychological experience of this population. Furthermore, caution is needed when extrapolating these findings and guiding optimal behaviors, given that a systematic evaluation to integrate and summarize the related research findings is lacking. To overcome this hurdle, a qualitative systematic synthesis is needed by summarizing the real perceptions and elucidating the feelings of lay responders performing CPR, which touch on the potential importance of providing more contextualized training based on the experience of actual responders, implementing targeted psychological interventions, and informing policy development for healthcare providers.

## Methods

### Study Design

The review was based on a synthesis of thematic findings from qualitative studies (PROSPERO ID: CRD42022360427). In this meta-synthesis, we performed thematic synthesis following the steps outlined by Sandelowski and Barroso. This review used the ENTREQ statement, which enhances the transparency framework for qualitative research reporting, to ensure the normative and transparent nature of the research findings [29].

### Search Strategy

We searched the following eight databases in September 2023: PubMed, Cochrane Library, Web of Science, OVID Medline, Embase, CINAHL, CNKI, and Wanfang. The search terms used were “qualitative research,” “Cardiopulmonary resuscitation,” and “First responder.” The search strategy for each database is described in Supplementary Appendix S1.

### Inclusion and Exclusion Criteria

We developed a sensitive and comprehensive search strategy based on the SPIDER tool (sample, phenomenon of interest, design, evaluation, research type) [30]. The inclusion criteria were as follows: lay responders who performed CPR; lay responders who had real experience performing CPR; original research published in English or Chinese; journal articles; and qualitative research. The exclusion criteria were as follows: quantitative studies, mixed studies, or reviews.

### Study Selection and Data Extraction

**FIGURE 1:**
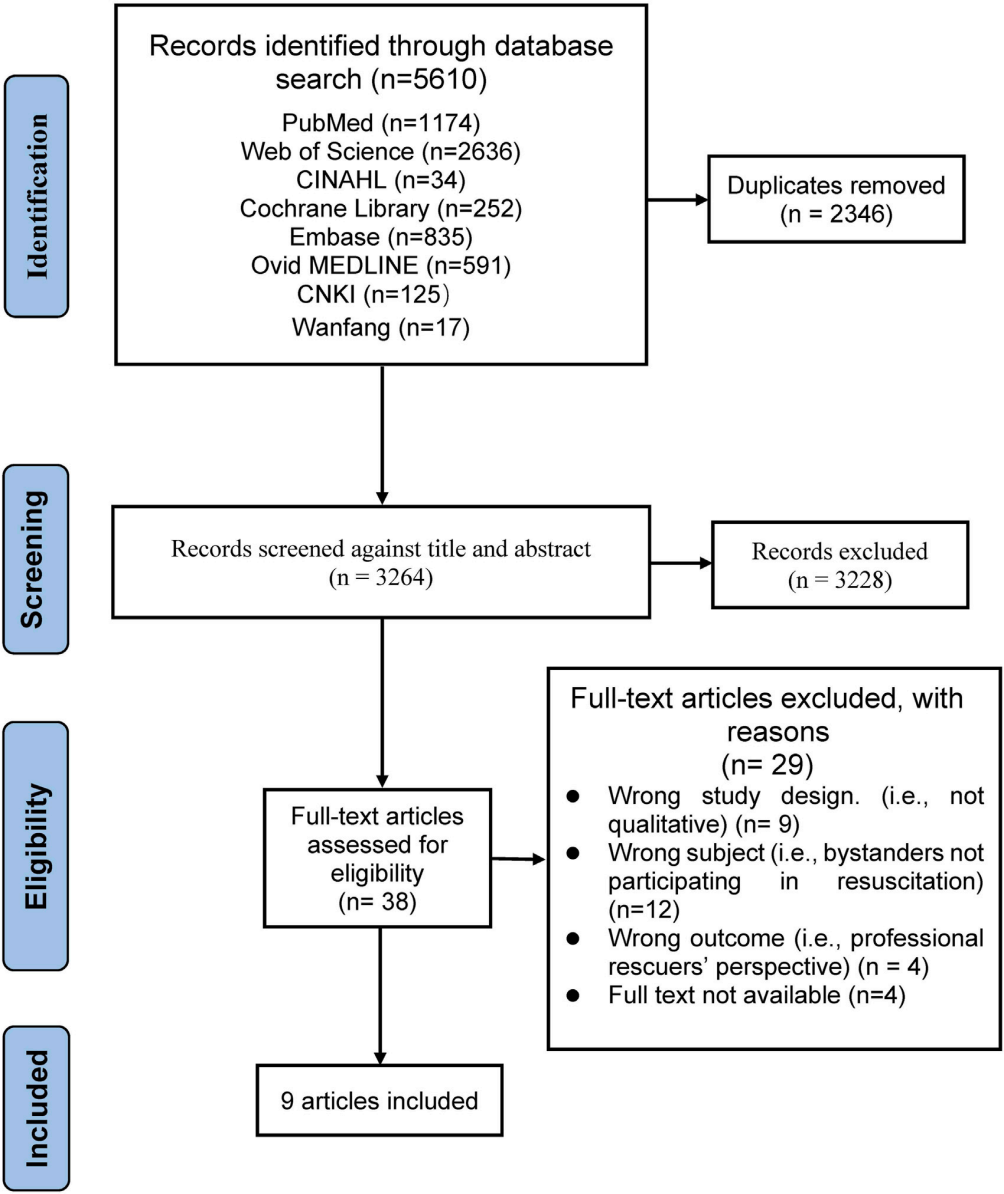
PRISMA flow chart of the search strategy results (China, 2023).

The search results were imported into Endnote X9, and duplicate studies were removed. Two reviewers independently screened the title, abstract, and full text (NL and CS), and disagreements were discussed and resolved by a third reviewer for inclusion (ZPC). We ultimately included nine studies with the following PRISMA flow chart (Figure 1). Two authors used Microsoft Excel to independently extract key information from the articles, including authors, year, country, sample, methods, study aims, and main findings. Disagreements were resolved through consultation and discussion. In addition, we independently extracted quotations from the articles for qualitative integration analysis.

### Appraisal of Methodological Quality

To ensure the quality of the study results, all studies were methodologically measured through the Critical Appraisal Skills Programme (CASP) [31]. Two researchers (NL and CS) independently measured the quality of each article, and disagreements were resolved through discussion with a third reviewer (WZ). The grade of quality assessment was not used as an inclusion or exclusion criterion for the literature in this study. The quality appraisal process is shown in Table 1.

**TABLE 1:** Results of the critical appraisal of the included studies (China, 2023).

	Axelsson [26] (2000)	Chen [32] (2020)	Hasselqvist [33] (2018)	Moller [16] (2014)	Mathiesen [34] (2016)	Mathiesen [35] (2017)	Mausz [27] (2018)	Skora [28] (2001)	Yang [36] (2022)
Item 1	Y	Y	Y	Y	Y	Y	Y	Y	Y
Item 2	Y	Y	Y	Y	Y	Y	Y	Y	Y
Item 3	Y	Y	Y	Y	Y	Y	Y	Y	Y
Item 4	Y	U	N	Y	Y	Y	Y	Y	Y
Item 5	Y	Y	Y	Y	Y	Y	Y	Y	Y
Item 6	N	U	U	U	U	N	N	U	N
Item 7	U	U	Y	Y	Y	Y	U	Y	Y
Item 8	Y	Y	Y	Y	Y	Y	Y	Y	U
Item 9	Y	Y	Y	Y	Y	Y	Y	Y	Y
Item 10	Y	Y	Y	Y	Y	Y	Y	Y	Y
Overall scores	8.5	8	8.5	9.5	9.5	9	8.5	9.5	8.5
Quality grade	Moderate to High	Moderate to High	Moderate to high	High	High	High	Moderate to High	High	Moderate to High

Noted: Y = Yes (1 point); N = No (0 points); U = Unclear (0.5 points). Item 1, Was there a clear statement of the aims of the research?; Item 2, Is a qualitative methodology appropriate?; Item 3, Was the research design appropriate to address the aims of the research?; Item 4. Was the recruitment strategy appropriate to the aims of the research?; Item 5, Was the data collected in a way that addressed the research issue?; Item 6, Has the relationship between the researcher and participants been adequately considered?; Item 7, Have ethical issues been taken into consideration?; Item 8, Was the data analysis sufficiently rigorous?; Item 9, Is there a clear statement of findings?; Item 10, Was this research valuable?

### Data Synthesis and Assessment

We used a three-stage thematic synthesis approach for the analysis, a process that was carried out by two researchers [37]: 1) using Microsoft Excel to code all quotes; 2) generating descriptive topics; and 3) developing analytical themes. We used the GRADE‐CERQual (Confidence in the Evidence from Reviews of Qualitative Research) approach to assess our confidence in each finding [38]. We assessed four components of methodological limitations [39], adequacy [40], coherence [41], and relevance [42]. All disagreements were resolved by discussion and confirmed by a third author (ZPC). The credibility assessment of the study results is presented in Supplementary Appendix S2 [43].

### Rigor, Trustworthiness, and Reflexivity

Our study illustrates this point by analysing the quotes from the original study. The research team members included nurses, librarians, research assistants and Red Cross members. Nurses and librarians were trained in qualitative research methods. During the study, team members communicated regularly in online meetings, and all disagreements were resolved through discussions. Finally, we presented the findings to lay responders who had experienced OHCA rescue and incorporated their recommendations into the final analytical themes.

## Results

### Characteristics of the Included Studies

These nine qualitative studies included 150 participants from six countries (Sweden, China, Norway, Denmark, Canada, United States) who participated in OHCA resuscitation. The characteristics of this study are shown in Table 2. We extracted a total of 207 quotes from the 9 included articles, synthesized 24 new findings according to the meaning of the introductions, and integrated 24 findings into 8 new categories and 4 integrated outcomes. All key supporting quotes per theme are shown in Supplementary Appendix S3.

**TABLE 2:** Characteristics of the included studies (China, 2023).

Authors/(Year)	Country	Participants	Methods	Study aims	Main findings
Axelsson et al., 2000 [26]	Sweden	N = 19, Aged 22–64, F/M, 11/8), Outcome: (S/D/U, 5/12/2)	Purposive sampling; focus group and in- dividual interviews; content analysis	To know more about the bystanders’ perceptions of their intervention	**Theme 1)** To have a sense of humanity **Theme 2)** To have competence **Theme 3)** To feel an obligation **Theme 4)** To have courage **Theme 5)** To feel exposed
Chen et al., 2020 [32]	China	N = 9, Aged 28–40, (F/M, 4/5), Outcome: (S, 9)	Semistructured interview; Grounded theory	Explore the experience of lay rescuers who had performed CPR and AED in public locations in Taiwan	**Theme 1)** Motivation **Theme 2)** Training reality discrepancy **Theme 3)** Psychological influence
Hasselqvist et al., 2018 [33]	Sweden	N = 22, Aged 23–54, (F/M, 5/17), Outcome: not reported	Purposive sampling; semistructured in-depth interviews; critical incident technique (CIT) and inductive qualitative content analysis	To explore firefighters’ and police officers’ experience of responding to OHCA in a dual dispatch	**Theme 1)** Preparedness **Theme 2)** Managing the scene **Theme 3)** The aftermath caring for rescuers
Moller et al., 2014 [16]	Danish	N = 33, (F/M, 16/17) Outcome: not reported	Semistructured interview; phenomenological method	To explore the concept of debriefing bystanders after participating in an OHCA resuscitation	**Theme 1)** Identification of OHCA. **Theme 2)** Emotional and perceptual experience with OHCA. **Theme 3)** Collaboration with healthcare professionals **Theme 4)** Patient outcome **Theme 5)** Reflexions
Mathiesen et al., 2016 [34]	Norway	N = 20, Aged 24–69, Outcome: (S/D/U, 14/5/1)	Snowball sampling; Semi structured in-depth interviews; content analysis	To explore reactions and coping strategies in lay rescuers who have provided CPR to OHCA.	**Theme 1)** Concern **Theme 2)** Uncertainty **Theme 3)** Coping strategies
Mathiesen et al., 2017 [35]	Norway	N = 10, Aged 24–69, (F/M, 4/6) Outcome: not reported	Semistructured interviews; content analysis	To gain a better understanding of why barriers to providing CPR are overcome	**Theme 1)** Valuing life itself **Theme 2)** Comprehension **Theme 3)** Normative obligation **Theme 4)** Confidence **Theme 5)** Context-specific CPR.
Mausz et al., 2018 [27]	Canada	N = 12, Aged 24–65, (F/M, 4/8) Outcome: not reported	Semistructured in-depth interviews and focus group; constructivist grounded theory	What is the experience of bystanders who have attempted to resuscitate the victim of a sudden, out-of- hospital cardiac arrest?	**Theme 1)** Being called to act **Theme 2)** Taking action **Theme 3)** Making sense of the experience
Skora et al., 2001 [28]	United States	N = 12 Average age 45, (F/M, 2/10) Outcome: not reported	Purposive sampling	To examine the thoughts, feelings, and motivations of laypersons who have attempted to resuscitate a stranger	**Theme 1)** Description of the resuscitation attempt **Theme 2)** Thoughts and feelings on first seeing the person who required resuscitation **Theme 3)** Thoughts and feelings during the course of the resuscitation event **Theme 4)** Thoughts and feelings immediately after the resuscitation event **Theme 5)** Discussion of feelings after the resuscitation event **Theme 6)** What motivated providing lifesaving measures to a stranger **Theme 7)** Hesitations, barriers, and obstacles to providing lifesaving measures **Theme 8)** Responses of prehospital providers to the respondents **Theme 9)** Recommendations for future CPR classes
Yang et al., 2022 [36]	China	N = 10, Aged 26–40, (F/M, 5/5) Outcome: not reported	Purposive sampling; semistructured in-depth interviews; content analysis	To explore the first responder experience of CPR.	**Theme 1)** First responders had a sense of responsibility and empathy **Theme 2)** First aid literacy of first responder needed to be improved **Theme 3)** First responder lacked professional first aid knowledge **Theme 4)** The first responder had heavy psychological pressure load during CPR, which was difficult to adjust **Theme 5)** The outcome of CPR will have far-reaching impact on the career achievement of the first responder

Noted: OHCA: out-of-hospital cardiac arrest; CPR: cardiopulmonary resuscitation; EMS: emergency medical services; BLS: basic life support; AED: automated external defibrillator; M/F: Male/Female; S/D/U: Survived/Died/Unknown.

### Emotional Ambivalence Before Cardiopulmonary Resuscitation

When faced with OHCA, lay responders may experience a variety of reactions, such as stress and panic in the face of unknown events, ambivalence about the need for help, and helplessness when unable to judge the patient’s condition, which are closely related to the respondents’ proficiency in CPR, previous experience performing CPR, and awareness of the importance of CPR. Of course, strong psychological quality and self-confidence, as well as encouragement and support from people around them, are also indispensable.

#### Pre-resuscitation Reaction

OHCA is sudden and unprepared, which may create panic among lay responders who do not know what is going on and who feel overwhelmed, especially for people who have not previously been involved in CPR.

*“Having somebody go down like that, it’s the first time I have ever seen something like that. It was like “Oh [expletive]! What is going on?”* [27]

*“She was lying on her back. And was not well, that was obvious. She was not conscious, but there was a sort of breathing. It was quite obvious she was really not well.”* [16]

When other people are present at the scene of an accident, lay responders may think that someone else may be able to help instead. The main reason may be the lack of self-confidence of lay responders, who are worried that their irregular rescue measures may cause greater harm to patients.

*“I wondered if I should get involved and was there a possibility that I could make the situation worse. I looked around to see if anyone was making a move to help him no one was.”* [28]

Of course, there are some lay responders who know the importance of racing against the clock to save patients’ lives. They help patients as much as they can by shouting for help, calling 911, or conducting a pre-CPR assessment.

*“[I thought] this guy needs help; activate 911. He's unconscious and pale. Check for breathing and pulse.”* [28]

#### Factors Influencing Cardiopulmonary Resuscitation Implementation

Factors such as lay responders’ knowledge, psychological readiness, values, and perceptions of outcomes can influence their behaviors. For responders who have attended training, training can be methodical. In addition, the family and friends of lay responders can affect their decisions in the face of patients’ need for CPR.

*“At the time, I felt really calm. I knew exactly what to do. It was very fortunate, because first I had done the course and after that the revision course, which I had done quite recently, only a few months earlier.”* [26]

*“When I was young, my family and friends affected my thought and behavior very much.”* [32]

### Psychological Tolerance During Cardiopulmonary Resuscitation

For lay responders, after overcoming the fear of starting resuscitation, there are still many difficulties in the process of resuscitation, which mainly include unreliable knowledge of CPR, learning theoretical knowledge without actual participation, or affecting the operation of resuscitation due to stress and tension. In addition, because of possible blood or body fluid exposure and the unknown condition of the patient’s disease, bystander respondents also considered their own safety or prevention of infection. In this case, support from professional rescue workers, respiratory balloon assistance instead of mouth-to-mouth artificial respiration or performing hands-only CPR can help lay responders better complete the rescue of patients.

#### Psychological Conflicts in Complex Situations

The respondents were confused about chest compressions. Most respondents were confused about the location and intensity of the compressions.

*“But I figured anywhere around the chest is a good thing.”* [27]

In particular, if an accident occurs during compressions, such as a broken rib, this can leave the responder uncertain about his or her actions and unsure of the next step to take.

*“When I heard a rib crack, then I wondered if I was doing it correctly, but still I went on.”* [26]

The automated external defibrillator (AED) has a built-in voice prompting system, so it is only necessary to follow the step-by-step instructions in the use of the AED, but there are still problems in the use of the AED by lay responders.

*“Others tended to misinterpret the prompts from the defibrillator, thinking the beeping metronome meant that they were performing compressions incorrectly.”* [27]

Some responders are concerned about spreading infectious diseases after artificial respiration to strangers, and they consider cleaning their own mouths to be a necessary protective behavior.

*“When we arrived at the hospital, I cleaned my mouth in the washroom because he was a stranger. Cleaning the mouth is a necessary behavior.”* [32]

#### Desire to Be Assisted by Others

Lay responders crave support and assistance in delivering life-saving treatment. The online dispatcher directing the lay responders to perform the operation made them feel in control, overcoming inner fears and calming down.

*“For one thing, you do not feel so alone; and you have a voice that gives you instructions without increasing the intensity by even a small amount*.”(42)

At the same time, lay respondents also said that support from people around them would give them strength.

*“One person told me that I ought to compress the chest continuously. I had the courage to keep chest compressions.”* [32]

The respondents were eager to receive support from medical personnel and positive comments about their behaviors, which would alleviate their internal uncertainty.

*“It was very nice that the physician calmly talked to the people involved, while the other staff was packing up the patient.”* [16]

### Perceived Experience After Cardiopulmonary Resuscitation

After lay responders perform CPR, they may try to understand the patient’s prognosis to express their concern for the patients and assess the quality of their CPR. This may have different impacts. On the one hand, the successful rescue of patients will increase the self-confidence of lay responders and strengthen the courage to carry out CPR again; on the other hand, the experience of failure rescue may increase the likelihood of long-standing psychological sequelae, such as self-doubt, guilt, and psychological fear in lay responders.

#### Post-resuscitation Perceptions

Regardless of whether the results were good or bad, lay respondents were eager to obtain results, believing it was their right. Lay responders were happy that the patients survived and were confident about performing bystander CPR next time.

*“I wondered about the outcome. I looked in the newspaper … …. Did we manage this, or did we not?”* [34]

However, if the patient has a poor prognosis or dies, they will doubt their own ability, wonder why they failed, and experience negative emotions as a result.

*“When I heard word [that] he passed, that was hard … …. Is it because I did not run fast enough? Was I supposed to start the process?”* [27]

Responders feel confident about helping again after experiencing real-life resuscitation, and they will be more confident and quicker to initiate resuscitation procedures in similar scenarios.

*“I believe I can make a quicker decision to start CPR next time.”* [32]

#### Subsequent Impact

Because OHCA usually occurs when the respondent is unprepared, it is an unexpected and frightening experience for the respondent, who may experience physiological reactions such as nightmares, vomiting, and loss of appetite following the rescue.

*“I felt terrible. Plus, I was throwing up. I probably saw the victim’s face in my dreams for a month.”* [28]

Of course, some respondents experienced psychological problems, such as depression, anxiety, and situational flashbacks. These psychological problems are especially prominent when they pass through the place of the incident.

*“I cannot go in the room [where the incident occurred]; I cannot even look at that door because my anxiety goes through the roof.”* [27]

### Enhancing Psychological Resilience

After providing CPR, some lay responders choose to ease psychological stress after OHCA through self-adjustment, self-relaxation or increased communication with family and friends. Of course, some lay responders consulted medical staff or psychological counsellors to seek more systematic guidance and help. In addition, empathy from other lay responders who have the same experience is also important. Psychotherapy is not achieved overnight and requires long-term maintenance and enhancement of the psychological resilience of lay responders.

#### Coping Strategies

Lay responders experience different physical and psychological reactions after performing CPR. They will adopt different coping strategies to return their lives to normal. In general, talking with others can make them feel calm and understood.

*“I spoke to my mother-in-law about it. I just told her what happened. I just had to calm down and so on.”* [16]

Some respondents opted for a self-acceptance approach to self-adaptation, and they began to think positively about the relationship between life and death.

*“I’ve got the answers. I’ve got the information I needed. I’ve had the rest, now it’s time to move forward.”* [27]

At the same time, respondents expressed the desire for psychological support after recovery and the idea of receiving medical staff consultation, whether in the short term or long term, which seems to be crucial for respondents.

*“It was a kind of first aid to me, and I liked that. Because when standing in the middle of it all and experiencing all that, the ambulance crew have to help other people. But no one helped me.”* [16]

The respondents expressed the importance of communicating with healthcare professionals, and this communication can enhance lay responders’ skills, increase confidence in potentially performing CPR in the future, and increase positive perceptions of their own performance.

*“I think it was nice to talk to a professional who knows what it is about 100 percent.”* [16]

#### Previous Experience Performing Cardiopulmonary Resuscitation

Lay responders offer some suggestions: bridging the gap between training and the reality of recovery, focusing on the physical and mental health of the respondent. In addition, Lay responders suggested adding the importance of sharing the real-life experience of rescuers to training, as this experience may help trainees mentally prepare for the actual implementation of CPR and enhance coping strategies.

*“If you are trained but cannot use your skills, then the training is meaningless. You could consider preparing people on how surrealistic a situation it is.”* [16]

After resuscitation, it is important to provide appropriate support to lay responders. This can include actively communicating with them after they have provided first aid, paying attention to their mental health, and taking steps to prevent psychological distress.

*“Stressing the spiritual and philosophical nature of death and the importance of speaking with someone after the experience.”* [28]

Lay responders also expressed their views on the training courses. The frequency of courses should be increased, sufficient practice and feedback opportunities for trainees should be provided, and workplace employee training should be increased.

*“Lower student-to-teacher ratio, lots of practice time and feedback opportunities.”* [28]

## Discussion

This study identified, reviewed, and synthesized nine qualitative articles that systematically explored the experience of lay responders performing CPR, and 4 analytical themes were generated: emotional ambivalence before CPR; psychological tolerance during CPR; perceived experience after CPR; and enhancing psychological resilience. Our findings highlight the importance of improved CPR training and follow-up social support for lay responders. The results of this study show that lay responders experience a series of emotional reactions before CPR and make complex decisions immediately under stressful conditions, which is consistent with the findings of previous studies [44, 45]. Our review suggested that the most common emotional reaction of lay responders to CPR was panic. Therefore, patients exhibit certain characteristics that may exacerbate the emotional reactions of lay responders. This can be attributed to the lack of experience and psychological preparation for lay responders performing CPR, as some emergency responders may experience psychological stress reactions due to past traumatic events, which can also affect their performance in CPR [46]. There is often discomfort associated with initiating CPR due to concerns about inappropriate or unethical resuscitation attempts. This can affect individuals’ willingness to perform CPR even after receiving training.

In addition to the emotional reactions of lay responders, the results also showed the “bystander effect,” where the presence of others makes lay responders hesitate to provide assistance, and they experience conflicting psychological reactions [47]. Interestingly, some responders perceive the presence of others as moral support, especially when people know each other, which reduces the indifferent attitude of lay responders [48]. In a study designed to investigate bystander witness type and receipt of bystander CPR [49], the investigators included 10,016 OHCA patients and showed that nonfamily witnessed OHCA patients were less likely to receive bystander CPR, confirming that witness type may have a potential impact. Indeed, the majority of OHCAs occur in residential areas or at home [50, 51]. Based on this, capitalizing on the bystander effect and conducting home-centered CPR training would be beneficial for improving the response rate of lay responders to family members in potential cardiac arrest and promoting survival from OHCA in residences. In addition, bystander indifference is caused by reflexive emotional reactions depending on the personality of the bystander [52]. This indicates that current training courses focus on skills and may not adequately prepare lay responders psychologically. It is worth noting that personality is also influenced by factors in the cultural environment of the East and West. Based on the results of the literature we included, it was shown that Easterners have conservative personalities, which may influence them to initiate bystander CPR in cases of uncertainty or lack of confidence [34], whereas Westerners are bold, enthusiastic and risk-taking, and are more likely to take action when they have some knowledge base [16]. Future training courses should consider the cognitive and complex emotional processes based on diverse cultural settings that occur during OHCA and expand the design of integrated emotional, motivational, and personality training interventions.

In the same vein, the possible knowledge curse in CPR training should not be ignored [53]. The curse of knowledge refers to the tendency for individuals to be influenced by their own knowledge and experience, which can lead to biases when attempting to understand or appreciate perspectives that are more naive or uninformed [54]. The reason for the “curse of knowledge” among CPR trainers is based on the tendency of others to share the same level of understanding and skills as oneself. Actually, the complexity and technical nature of CPR training require a high level of proficiency from instructors, whereas there is a significant gap between theoretical knowledge and practical skills retention among lay responders. First, lay responders find it difficult to identify signs of cardiac arrest, which may lead to delayed CPR. Studies have shown that the inability to identify a patient’s blueness, abnormal breathing, and vital signs may be obstacles for lay responders [55]. This is consistent with our integration results. In addition, lay responders reported various difficulties during the resuscitation process, such as problems with the location and force of chest compressions. The reasons for these difficulties may be the lack of proficiency of the trainer, which causes excessive tension in real situations and leads to the failure of technical points, or the inability of simulation training to simulate multiple emergency situations, resulting in the inability to respond to various emergencies. The key to maintaining skills depends on the frequency and timing of retraining, and regular CPR training every 6 months is recommended to ensure that lay responders maintain their practical CPR skills [56]. In simplified terms, the knowledge curse in CPR training can be mitigated by ensuring regular and timely retraining, employing effective teaching methods that focus on practical skills, and addressing attitudes towards CPR to encourage its use in emergency situations.

During CPR, strong psychological tolerance plays a pivotal role in effective resuscitation, which is susceptible to theoretical knowledge, practice, patient condition and surroundings. In addition, loss aversion may touch on the potential importance of performing CPR. Loss aversion, as discussed in the provided evidence, refers to the psychological phenomenon where individuals exhibit a strong dislike or fear of loss, particularly when it involves significant aspects of their identity or wellbeing [57]. The psychological impact of loss aversion can affect how lay responders perceive and manage their own reactions to CPR scenarios. For instance, the fear of losing a patient can lead to increased stress and anxiety among clinicians, which might affect their performance during CPR. This internal conflict between the desire to save lives and the fear of causing unnecessary harm or loss can complicate the resuscitation process. Loss aversion in the context of CPR involves a complex interplay of psychological factors that influence patient and provider behaviors and decisions. Understanding these dynamics is crucial for improving communication strategies and providing more effective support during critical care situations. It also highlights the need for comprehensive training and support systems for healthcare providers to help lay responders manage their reactions to potential losses and make informed decisions that align with both ethical standards and patient safety [58].

Of note, early defibrillation during CPR is one of the key factors in improving the prognosis of patients with OHCA [59, 60]. The results of an observational study based on 8,269 patients with OHCA showed that for OHCA occurring in public, patients had the highest probability of surviving to hospital discharge if they received both bystander-initiated CPR and defibrillation performed by a bystander (OR 4.33, 95% CI 2.11–8.87) [61]. However, many lay responders seem to have some psychological fear and lack experience using AEDs. In the included literature, two papers elucidated the barriers to the use of AEDs among lay responders, highlighting the potential importance of AED training in basic life support courses [62]. Previous studies have shown that the correct use of AEDs does not require specific skills [63], only following voice prompts. This encourages untrained lay responders to use AEDs [64]. Cardiac arrest is a rapidly evolving crisis situation in a dynamic environment that requires emergency personnel to perform highly technical interventions to save lives under emotional stress [65]. Current CPR training seems unable to cope with real-life resuscitation, and lay responders need more support in terms of both psychological preparation and technical training. It is recommended that contextualized CPR training be developed based on the real experience of lay responders, specifically by incorporating descriptions of the difficulties experienced by lay responders who prepare them for realistic recovery.

More importantly, after CPR, lay responders face diverse perceived experience, which should be prioritized in future studies. According to our integrated results, most lay responders actively adjusted their cognition and took positive action, whereas a proportion of lay responders have to face short-term psychological stress and self-doubt [27]. Moreover, the psychological impact of witnessing such a life-threatening event can lead to long-term psychological distress, including symptoms of PTSD, anxiety, and depression [66]. Social support plays a crucial role in mitigating these psychological impacts. Studies have shown that perceived social support is strongly associated with better recovery outcomes following trauma or illness [67]. In the context of cardiac events, both the presence of social support and the quality of this support are critical factors that influence the psychological wellbeing of individuals involved [68]. An alternative approach is that the coping strategies of lay responders are in line with stress coping theory [69]. Coping theory defines coping as an effort by an individual to deal with demands from the environment, with the aim of making these demands tolerable and reducing stress and conflict [69]. This theory provides guidance and a reference for individuals to cope with stressful events, including the psychological stress experienced by lay responders after performing CPR. According to this theory, individuals can take the following measures to cope with such stress: first, they can actively adjust their cognition by practicing positive thinking and reinterpreting the event to reduce the impact of negative emotions; second, they can seek external information and professional support and help, such as communicating with family, friends, and professionals for advice and support; and finally, they can take positive actions, such as participating in psychological counselling, physical exercise, and relaxation training activities, to alleviate psychological stress. Taken together, we need to pay more attention to the life status of lay responders and provide them with personalized psychological counselling services and follow-up system support.

### Limitations

Some limitations of this study should not be ignored. First, selection bias is unavoidable due to the reason that not all lay CPR rescuers are willing to recall and participate in research, and usually, the subjects who can be interviewed are those who have succeeded in rescue or who have a positive attitude. These biases can influence the integrity of the integrated findings. Second, we did not account for the interaction between lay responders and professional medical responders, which may potentially influence the performance and response of lay responders. Future research should consider incorporating variables that reflect the relationship dynamics between these groups to better understand their impact on emergency response outcomes. In addition, few studies included in our analysis noted the time interval between performing CPR and collecting qualitative data, which may have introduced recall bias and affected the results. Finally, qualitative research is deeply embedded in specific cultural and contextual settings. Integrating findings across different cultures or contexts can be challenging due to varying social norms, values, and behaviors.

### Conclusion

This study systematically assessed real experience of lay responders performing CPR. Our results emphasize that, in addition to attaching importance to CPR training, healthcare professionals should fully understand the real experience of lay responders and promptly intervene in possible psychological sequelae to improve the response rate of lay responders and promote public health. As noted, our knowledge of lay responders’ perceptions of performing CPR can compensate for biases in medical research regarding treatment outcomes, clinical providers, and decision-making by reflecting the true perspective of lay responders’ experience performing CPR. Additionally, the emotional and psychological impacts of CPR, which are susceptible to “loss aversion” and “bystander effects,” as well as the “knowledge curse” during CPR implement and training, can also affect the enthusiasm of lay responders to perform effectively in future incidents. Not surprisingly, these findings support the emerging consensus that incorporating these factors into our analysis could provide a more comprehensive understanding of the psychological dynamics of lay responders throughout the CPR process.
