# Supplementary material for: The Real Experience of Lay Responders Performing Cardiopulmonary Resuscitation: A Synthesis of Qualitative Evidence
Source: Public Health Rev. 2024 Jun 5;45:1606650. doi: 10.3389/phrs.2024.1606650 (PMC11188311; doi:10.3389/phrs.2024.1606650)
Supplement: Supplementary file 1 [file DataSheet1.zip › Appendix 1.DOCX]

**Appendix 1 search strategy**

| **PubMed** | | Total |
| --- | --- | --- |
| #1 | (“Cardiopulmonary Resuscitation"[Mesh]) OR (Cardiopulmonary resuscitation) | 31211 |
| #2 | (((((((First responder) OR ("Emergency Responders"[Mesh])) OR (Emergency Responders)) OR (bystander)) OR (witness)) OR (layperson)) OR (lay rescuers)) OR (lay responders) | 72472 |
| #3 | (("Adaptation, Psychological"[Mesh]) OR Adaptation, Psychological OR Perspective OR experience OR feeling OR opinion OR satisfaction OR dissatisfaction OR needs OR demands OR perceptions OR expectations OR attitudes OR Coping strategies) OR coping OR coping OR behaviour OR behaviour OR behaviour mechanism | 7764672 |
| #4 | #1 AND #2 AND #3 | **1174** |

| **Web of science** | | Total |
| --- | --- | --- |
| #1 | TS=(Cardiopulmonary resuscitation OR Resuscitation OR Mouth-to-Mouth OR Mouth-to-Mouth Resuscitations OR Mouth-to-Mouth Resuscitation OR Resuscitations OR Mouth-to-Mouth OR Mouth to Mouth Resuscitation OR Cardio-Pulmonary Resuscitation OR CPR OR Resuscitation, Cardiopulmonary OR Cardio Pulmonary Resuscitation OR Resuscitation, Cardio-Pulmonary OR Basic Cardiac Life Support OR Life Support, Basic Cardiac OR Code Blue) | 68126 |
| #2 | TS=(First responder OR Emergency Responder OR Responder, Emergency OR Responders, Emergency OR Emergency First Responders OR Emergency First Responder OR First Responder, Emergency OR First Responders, Emergency OR First Responders OR First Responder OR Responder, First OR Responders, First OR bystander OR witness OR layperson OR lay rescuers OR lay responders) | 82926 |
| #3 | TS=(Perspective OR experience OR feeling OR opinion OR satisfaction OR dissatisfaction OR needs OR demands OR perceptions OR expectations OR attitudes OR Coping strategies OR coping OR coping OR behaviour OR behaviour OR behaviour mechanism OR Adaptation, Psychologic OR Psychologic Adaptation OR Psychological Adaptation OR Adjustment OR Coping Behavior OR Behavior, Coping OR Behaviors, Coping OR Coping Behaviors OR Coping Skills OR Coping Skill OR Skill, Coping OR Skills, Coping OR Coping Strategies OR Coping Strategy OR Strategies, Coping OR Strategy, Coping OR Behavior, Adaptive OR Adaptive Behavior OR Adaptive Behaviors OR Behaviors, Adaptive) | 8851661 |
| #4 | #1 AND #2 AND #3 | **1,539** |

| **Cochrane Library** | | Total |
| --- | --- | --- |
| #1 | Cardiopulmonary resuscitation OR Resuscitation OR Mouth-to-Mouth OR Mouth-to-Mouth Resuscitations OR Mouth-to-Mouth Resuscitation OR Resuscitations OR Mouth-to-Mouth OR Mouth to Mouth Resuscitation OR Cardio-Pulmonary Resuscitation OR CPR OR Resuscitation, Cardiopulmonary OR Cardio Pulmonary Resuscitation OR Resuscitation, Cardio-Pulmonary OR Basic Cardiac Life Support OR Life Support, Basic Cardiac OR Code Blue | 9113 |
| #2 | **First responder OR Emergency Responder OR Responder, Emergency OR Responders, Emergency OR Emergency First Responders OR Emergency First Responder OR First Responder, Emergency OR First Responders, Emergency OR First Responders OR First Responder OR Responder, First OR Responders, First OR bystander OR witness OR layperson OR lay rescuers OR lay responders** | 290274 |
| #3 | **Perspective OR experience OR feeling OR opinion OR satisfaction OR dissatisfaction OR needs OR demands OR perceptions OR expectations OR attitudes OR Coping strategies OR coping OR coping OR behaviour OR behaviour OR behaviour mechanism OR Adaptation, Psychologic OR Psychologic Adaptation OR Psychological Adaptation OR Adjustment OR Coping Behavior OR Behavior, Coping OR Behaviors, Coping OR Coping Behaviors OR Coping Skills OR Coping Skill OR Skill, Coping OR Skills, Coping OR Coping Strategies OR Coping Strategy OR Strategies, Coping OR Strategy, Coping OR Behavior, Adaptive OR Adaptive Behavior OR Adaptive Behaviors OR Behaviors, Adaptive** | 12846 |
| #4 | #1 AND #2 AND #3 | **252** |

| **Embase** | | Total |
| --- | --- | --- |
| #1 | **ti，ab，kw (Cardiopulmonary resuscitation OR Resuscitation OR Mouth-to-Mouth OR Mouth-to-Mouth Resuscitations OR Mouth-to-Mouth Resuscitation OR Resuscitations OR Mouth-to-Mouth OR Mouth to Mouth Resuscitation OR Cardio-Pulmonary Resuscitation OR CPR OR Resuscitation, Cardiopulmonary OR Cardio Pulmonary Resuscitation OR Resuscitation, Cardio-Pulmonary OR Basic Cardiac Life Support OR Life Support, Basic Cardiac OR Code Blue)** | 109635 |
| #2 | **ti，ab，kw (****First responder OR Emergency Responder OR Responder, Emergency OR Responders, Emergency OR Emergency First Responders OR Emergency First Responder OR First Responder, Emergency OR First Responders, Emergency OR First Responders OR First Responder OR Responder, First OR Responders, First OR bystander OR witness OR layperson OR lay rescuers OR lay responders)** | 30385 |
| #3 | **ti，ab，kw (Perspective OR experience OR feeling OR opinion OR satisfaction OR dissatisfaction OR needs OR demands OR perceptions OR expectations OR attitudes OR Coping strategies OR coping OR coping OR behaviour OR behaviour OR behaviour mechanism OR Adaptation, Psychologic OR Psychologic Adaptation OR Psychological Adaptation OR Adjustment OR Coping Behavior OR Behavior, Coping OR Behaviors, Coping OR Coping Behaviors OR Coping Skills OR Coping Skill OR Skill, Coping OR Skills, Coping OR Coping Strategies OR Coping Strategy OR Strategies, Coping OR Strategy, Coping OR Behavior, Adaptive OR Adaptive Behavior OR Adaptive Behaviors OR Behaviors, Adaptive)** | 3154221 |
| #4 | #1 AND #2 AND #3 | **835** |

| **CINAHL** | | Total |
| --- | --- | --- |
| #1 | **SU (****Cardiopulmonary resuscitation OR Resuscitation OR Mouth-to-Mouth OR Mouth-to-Mouth Resuscitations OR Mouth-to-Mouth Resuscitation OR Resuscitations OR Mouth-to-Mouth OR Mouth to Mouth Resuscitation OR Cardio-Pulmonary Resuscitation OR CPR OR Resuscitation, Cardiopulmonary OR Cardio Pulmonary Resuscitation OR Resuscitation, Cardio-Pulmonary OR Basic Cardiac Life Support OR Life Support, Basic Cardiac OR Code Blue)** | 18239 |
| #2 | **SU (****First responder OR Emergency Responder OR Responder, Emergency OR Responders, Emergency OR Emergency First Responders OR Emergency First Responder OR First Responder, Emergency OR First Responders, Emergency OR First Responders OR First Responder OR Responder, First OR Responders, First OR bystander OR witness OR layperson OR lay rescuers OR lay responders)** | 2226 |
| #3 | **SU (****Perspective OR experience OR feeling OR opinion OR satisfaction OR dissatisfaction OR needs OR demands OR perceptions OR expectations OR attitudes OR Coping strategies OR coping OR coping OR behaviour OR behaviour OR behaviour mechanism OR Adaptation, Psychologic OR Psychologic Adaptation OR Psychological Adaptation OR Adjustment OR Coping Behavior OR Behavior, Coping OR Behaviors, Coping OR Coping Behaviors OR Coping Skills OR Coping Skill OR Skill, Coping OR Skills, Coping OR Coping Strategies OR Coping Strategy OR Strategies, Coping OR Strategy, Coping OR Behavior, Adaptive OR Adaptive Behavior OR Adaptive Behaviors OR Behaviors, Adaptive)** | 515920 |
| #4 | #1 AND #2 AND #3 | **34** |

| **MEDLINE** | | Total |
| --- | --- | --- |
| #1 | **(Cardiopulmonary resuscitation OR Resuscitation OR Mouth-to-Mouth OR Mouth-to-Mouth Resuscitations OR Mouth-to-Mouth Resuscitation OR Resuscitations OR Mouth-to-Mouth OR Mouth to Mouth Resuscitation OR Cardio-Pulmonary Resuscitation OR CPR OR Resuscitation, Cardiopulmonary OR Cardio Pulmonary Resuscitation OR Resuscitation, Cardio-Pulmonary OR Basic Cardiac Life Support OR Life Support, Basic Cardiac OR Code Blue).mp** | 89411 |
| #2 | **First responder OR Emergency Responder OR Responder, Emergency OR Responders, Emergency OR Emergency First Responders OR Emergency First Responder OR First Responder, Emergency OR First Responders, Emergency OR First Responders OR First Responder OR Responder, First OR Responders, First OR bystander OR witness OR layperson OR lay rescuers OR lay responders** | 22230 |
| #3 | **Perspective OR experience OR feeling OR opinion OR satisfaction OR dissatisfaction OR needs OR demands OR perceptions OR expectations OR attitudes OR Coping strategies OR coping OR coping OR behaviour OR behaviour OR behaviour mechanism OR Adaptation, Psychologic OR Psychologic Adaptation OR Psychological Adaptation OR Adjustment OR Coping Behavior OR Behavior, Coping OR Behaviors, Coping OR Coping Behaviors OR Coping Skills OR Coping Skill OR Skill, Coping OR Skills, Coping OR Coping Strategies OR Coping Strategy OR Strategies, Coping OR Strategy, Coping OR Behavior, Adaptive OR Adaptive Behavior OR Adaptive Behaviors OR Behaviors, Adaptive** | 2534543 |
| #4 | #1 AND #2 AND #3 | **591** |

| **CNKI** | | Total |
| --- | --- | --- |
| #1 | 篇关摘（心肺复苏 + 人工呼吸 + 生命支持 + 复苏 + CPR ） | 132322 |
| #2 | 篇关摘（第一反应者 + 第一响应者 + 旁观者 + 目击者 + 外行人救援者 + 外行人反应者 + 紧急救援人员） | 12056 |
| #3 | 篇关摘（观点 + 经验 + 感觉 + 需求 + 态度 + 看法 + 应对 + 心理） | 9,365,369 |
| #4 | #1 AND #2 AND #3 | **71** |

| **WANFANG** | | Total |
| --- | --- | --- |
| #1 | 题名或关键词（心肺复苏 or 人工呼吸 or 生命支持 or 复苏 or CPR） | 79878 |
| #2 | 题名或关键词（第一反应者 or 第一响应者 or 旁观者 or 目击者 or 外行人救援者 or 外行人反应者 or 紧急救援人员） | 8333 |
| #3 | 题名或关键词（观点 or 经验 or 感觉 or 需求 or 态度 or 看法 or 应对 or 心理） | 5244103 |
| #4 | #1 AND #2 AND #3 | **17** |
