# Supplementary material for: The Real Experience of Lay Responders Performing Cardiopulmonary Resuscitation: A Synthesis of Qualitative Evidence
Source: Public Health Rev. 2024 Jun 5;45:1606650. doi: 10.3389/phrs.2024.1606650 (PMC11188311; doi:10.3389/phrs.2024.1606650)
Supplement: Supplementary file 1 [file DataSheet1.zip › Appendix 2.DOCX]

**Supplementary file 2 GRADE-CERQual Assessment Results**

| **Finding number** | **Review finding** | **Studies contributing to the review finding** | **CER Qual assessment** | **Explanation of Confidence in the Evidence Assessment** |
| --- | --- | --- | --- | --- |
| ***Theme1 Emotional ambivalence before CPR*** | | | | |
| **Subtheme 1** **Pre- resuscitation reaction** | | | | |
| 1 | **Lose their heads in panic:** Participants were surprised by what they encountered, and they experienced a variety of emotions, such as nervousness, panic, and awfulness. | **5 studies** – 3, 6, 7, 8, 9 | Moderate | Due to minor concerns regarding coherence, relevance and adequacy, and moderate concerns regarding methodological limitations |
| 2 | **Feeling conflicted:** Lay responders felt conflicted about whether to rescue victims and were unsure whether their actions were effective. | **3 studies** – 4, 8, 9 | Low | Due to minor concerns regarding methodological limitations and coherence, and moderate concerns regarding relevance and adequacy |
| 3 | **Unable to judge:** Lay responders have difficulty determining the indications for starting CPR, and they are confused about what is happening to the resuscitated subject. | **4 studies** – 4, 6, 7, 8 | Moderate | Due to minor concerns regarding methodological limitations, relevance and coherence, and moderate concerns regarding adequacy |
| 4 | **Take immediate action:** Some responders don't have time to think and take immediate action to try to save a person in out-of-hospital cardiac arrest. | **2 studies** – 7, 8 | Low | Due to minor concerns regarding coherence, moderate concerns regarding methodological limitations, and serious concerns regarding relevance and adequacy |
| **Subtheme 2 Factors influencing CPR implementation** | | | | |
| 5 | **Subjective factors:** Subjective factors that influence whether lay responder performs CPR include knowledge, preparation, values, and perception of the outcome. | **8 studies –** 1, 2, 3, 4, 6, 7, 8, 9 | High | Due to minor concerns regarding methodological limitations, coherence and relevance, adequacy |
| 6 | **Objective factors:** Objective factors that influence lay responders to perform CPR include: work reasons, knowledge of first aid techniques, influence of family and friends, religion and possession of training certificates | **5 studies** – 2, 4, 7, 8, 9 | Moderate | Due to minor concerns regarding methodological limitations, coherence, and adequacy, and moderate concerns regarding relevance |
| ***Theme 2 Psychological tolerance during CPR*** | | | | |
| **Subtheme 3 Psychological conflicts in complex situations** | | | | |
| 7 | **Desire for support and help from those around you:** lay responders crave the support of those around them, which makes them feel empowered, otherwise they feel that time passes slowly and they feel apathy. | 3 studies **–** 1, 2, 6 | Low | Due to minor concerns regarding coherence, and relevance, and moderate concerns regarding methodological limitations and adequacy |
| 8 | **Desire for professional support and recognition:** Guidance from online dispatchers and medical workers arriving at the scene gives lay responders a lot of guidance, which makes their behavior more confident. | 3 studies **–** 4, 6, 8 | Low | Due to minor concerns regarding coherence, moderate concerns regarding methodological limitations, and serious concerns regarding relevance and adequacy |
| **Subtheme 4** **Desire to be assisted by others** | | | | |
| 9 | **Ambivalence about resuscitation methods:** Participants were confused about resuscitation modalities, including compression sites, AED usage, and mouth-to-mouth respiration. | **5 studies** – 1, 2, 7, 8, 10 | Moderate | Due to minor concerns regarding methodological limitations, coherence and adequacy, and moderate concerns regarding relevance |
| 10 | **Confusion about the process:** Familiarity with the procedure may affect the resuscitator's emotions; if they are familiar with the process, they will feel calm, otherwise panic will set in. | **2 study –** 2, 7 | Low | Due to minor concerns regarding methodological limitations and coherence, and moderate concerns regarding relevance and adequacy |
| 11 | **Confusion over the outcome of the resuscitation:** Participants were unsure of the signs of successful resuscitation and considered the presence of breathing in the resuscitated subject to be a sign of survival. | **2 study** – 7, 8 | Low | Due to minor concerns regarding methodological limitations and coherence, and moderate concerns regarding relevance and adequacy |
| 12 | **Worrying about the consequences:** Participants have a variety of concerns during the operation, such as fear of contracting infectious diseases, fear of legal liability, fear of death of the resuscitator, etc. | **5 studies** – 1, 3, 7, 8, 9 | Moderate | Due to minor concerns regarding coherence, relevance and adequacy, and moderate concerns regarding methodological limitations |
| ***Theme 3 Perceived experience after CPR*** | | | | |
| **Subtheme 5** **Post- resuscitation perceptions** | | | | |
| 13 | **Focus on resuscitation outcomes:** Respondents were resuscitated in a variety of ways, such as reading the newspaper, seeing if the flag was lowered, receiving flowers, etc. | **3 studies** – 2, 4, 6 | Moderate | Due to minor concerns regarding methodological limitations, coherence, and relevance, and moderate concerns regarding adequacy |
| 14 | **The joy of a successful resuscitation:** Survival of the resuscitated subject will make the respondent feel happy, valuable and more confident. | **4 studies** – 2, 5, 8, 9 | Moderate | Due to minor concerns regarding methodological limitations, coherence and relevance, and moderate concerns regarding adequacy |
| 15 | **The frustration of a failed resuscitation:** Failure to recover can lead to guilt, feelings of powerlessness and doubts about one's own abilities. | **3 studies** – 1, 7, 8 | Low | Due to minor concerns regarding relevance and coherence, and moderate concerns regarding adequacy and methodological limitations |
| 16 | **Perceptions of resuscitation behaviour:** Cardiac arrest is very common, feeling certain that you have performed the act of resuscitation, having a clearer understanding of the resuscitation process and taking the resuscitation training course more seriously. | **5 studies** – 1, 2, 3, 4, 9 | Moderate | Due to minor concerns regarding coherence, relevance and adequacy, and moderate concerns regarding methodological limitations |
| **Subtheme 6** **Subsequent impact** | | | | |
| 17 | **Physiological reactions：**After the resuscitation act, the responders experience physiological reactions such as insomnia, nightmares, vomiting and loss of appetite. | **3 studies** – 5, 7, 8 | Moderate | Due to minor concerns regarding methodological limitations, coherence, and adequacy, and moderate concerns regarding relevance |
| 18 | **Psychological reactions：**Respondents experience psychological problems such as low mood, anxiety, situational flashbacks, etc. | **7 studies** – 1, 2, 3, 5, 6, 7, 9 | High | Due to minor concerns regarding methodological limitations, coherence and relevance, adequacy |
| ***Theme 4 Enhancing psychological resilience*** | | | | |
| **Subtheme 7 Coping strategies** | | | | |
| 19 | **Responding by Talking:** Talking to a family member, friend or colleague can be calming, and the more often you talk about it, the less it affects you. | **3 studies** – 2, 4, 8 | Low | Due to minor concerns regarding methodological limitations and coherence, and moderate concerns regarding relevance and adequacy |
| 20 | **Acceptance:** Accept what happened, accept their own behavior, after all, they have also worked hard. | **4 studies –** 3, 4, 7, 9 | Moderate | Due to minor concerns regarding methodological limitations and coherence, and moderate concerns regarding adequacy |
| 21 | **Support from medical staff:** Having medical attention and regular counselling after resuscitation allows the respondent to have a clearer perception of their resuscitation behaviour and reduces psychological stress. | **4 studies –** 2, 4, 5, 8 | Moderate | Due to minor concerns regarding coherence, relevance and adequacy, and moderate concerns regarding methodological limitations |
| **Subtheme 8 Previous experience performing CPR** | | | | |
| 22 | **Suggestions for training courses:** Resuscitators made their own suggestions for training courses to better suit the actual. | **2 studies** – 4, 8 | Low | Due to minor concerns regarding coherence and methodological limitations, and serious concerns regarding relevance and adequacy |
| 23 | **Closing the gap between training and reality:** The training did not seem to prepare them for the real act of resuscitation, and lay responders suggested that the training include more sharing of real resuscitation cases to prepare them for actual resuscitation. | **4 studies –** 4, 7, 8, 9 | Moderate | Due to minor concerns regarding methodological limitations and coherence, and moderate concerns regarding adequacy |
| 24 | **Focus on the physical and mental health of the respondent:** Follow-up psychological support and counselling is recommended for resuscitators to reduce the short and long-term effects of resuscitation behaviour on them. | **2 studies** – 4, 8 | Low | Due to minor concerns regarding coherence, moderate concerns regarding methodological limitations and serious concerns regarding relevance and adequacy |
