# Supplementary material for: The Real Experience of Lay Responders Performing Cardiopulmonary Resuscitation: A Synthesis of Qualitative Evidence
Source: Public Health Rev. 2024 Jun 5;45:1606650. doi: 10.3389/phrs.2024.1606650 (PMC11188311; doi:10.3389/phrs.2024.1606650)
Supplement: Supplementary file 1 [file DataSheet1.zip › Appendix 3.DOCX]

**Appendix 3 supporting quotes**

**Key supporting quotes per theme**

| **Theme** | **Subtheme** | **supporting quotes** |
| --- | --- | --- |
| 1. Emotional ambivalence before CPR | a. Pre-resuscitation reaction | - “I got it into my head that I would make him come back, I had that belief. I thought, I almost thought that I would make him come back.” - “Perhaps he had a chance to come back to life.” - “Yes, somebody was lying on the ground and needed help.” - “It was a bit unpleasant when I felt that phlegm coming up, which I had to deal with, of course it does, but the other thing takes over…the wish to help.” - “It was only natural, if I can help I’ll do it.” - “I don’t think that I thought anything at all, I just observed that it was an emergency, so I didn’t really think of anything else.” - “I didn’t think what I should or shouldn’t do. Not that I stopped and thought that now I’m going to do so and so, but all of a sudden I was just there.” - “I grabbed him so he didn’t hit the floor, and then I noticed that he was sweating and gasping for air. That was the first sign. As soon as I got a hold of him [I screamed] ‘Mark! Mark! Stay with me!” - “The way his eyes were, like a normal person’s eyes are not like that … Why? Why did his eyes look like that?” - “Having somebody go down like that, it’s the first time I have ever seen something like that. It was like “Oh [expletive]! What is going on?” - “And I went to him and I saw him and I saw his face and his body and I just [crying], thought for a second ‘What do I do?’ and I just ran so fast, and I didn’t even know how to call – I touch my phone all the time and I didn’t remember my password – and I just ran to the [office] door and I hit the door so hard and I screamed so loud and I said ‘Kristina! Kristina! Call 911 now!” - “That’s enough to throw someone into panic mode, like no one else stepped in, everyone else was just looking. But you’ve got to get past that.” - “It was 5:00 in the evening. I was going to dinner. I had my key out and was ready to put it in the Jock when I saw my coworker holding the victim on the side of the pool. I immediately ran to the pool to assist her. There were about IO peopie in the pool area.” - “I thought he was just out of breath.” - “[I thought] this guy needs help; activate 911. He's unconscious and pale. Check for breathing and pulse.” - “I've never seen anyone that blue.” - “I've never seen a dead guy before.” - “He weighed about 220 pounds and was sitting under his truck hood completely inside the engine compartment [The] first thing I saw was his blue feet (barefooted), and I figured he had a heart attack because of his age and his pack of Marlboros in his shirt.” - “I was a little indecisive, my mind was blank, and I did not know where to start, I studied not medicine, usually online occasionally seeing the scene of first aid, and then I know some first aid knowledge, the specific operation of words we do not have much experience, and not professional, see how others do how to do it, is to call him in the past, he did not answer me, I used my hands to try to see, there is no respiration, I began to give him heart compressions, next to someone to call an ambulance, from this incident to learn the first aid method is too important.” - “I did a moment of psychological construction, I was also a little panicked because my first aid knowledge comes from my usual TV, brush TikTok and other occasional broadcasts of first aid small ads, I take the subway to and from work, but also see the car to promote first aid knowledge of the video, watched several times.” - “When I saw him on the floor, he’s not moving, he’s turning blue, something is clogging something, right?” - “I never imagined or thought of finding a person in cardiac arrest while I was outdoors jogging.” - “She was lying on her back. And was not well, that was obvious (. . .). She was not conscious, but there was a sort of breathing. It was quite obvious she was really not well.” - “It was a young girl. It just feels so damned awful and you know when you’ve got two girls of your own as well, it just feels awful…they had taken cocaine, amphetamine…” - “I don’t have any medical training; I could see that half of the brain was out, maybe it works anyway, I don’t have enough knowledge to decide.” - “One has to be prepared for what one might see. and that a person can look strange even if he or she hasn’t been dead for many hours. And that it is possible to be cold, even though you’re not really dead yet. And such basic things. You just have to go down on the floor and get started (with CPR), it should be very simple, you have to know that it may be disgusting.” - “Please live.” - “He was not going to make it.” |
|  | b. Factors influencing CPR implementation | - “At the time I felt really calm. I knew exactly what to do. It was very fortunate, because first I had done the course and after that the revision course, which I had done quite recently, only a few months earlier. It was so fresh in my mind then…” - “I had really no recollection of what to do.” - “I’ve never done this before nor have I experienced it.” - “We saw a video tape on the course…almost like an ‘aha’ experience when it happened, that I had almost prepared myself, I had actually imagined myself in that situation, I really had.” - “I’ve always been aware that it would happen.” - “I never though that it would happen to me. Then I went out with a person who was healthy, I didn’t give anything like that a thought, nor had she shown any signs…” - “It’s not something you expect to happen on an ordinary day at work, to be faced with a thing like that.” - “Something had to be done, I had to do something.” - “I saw their powerlessness and the panic, the fear in the children and grandchildren at seeing their grandfather sitting there and them not being able to reach him. It was terrible and everybody just stood there, looking, and I had to…” - “Had there been somebody else, I would willingly have left it to them, if it was somebody who really knew what to do, yes absolutely, but there was nobody and then I just had to tackle it.” - “Of course, when you’ve done a course like this and sort of have it confirmed or have papers to show that you have done it, then it’s perhaps expected of you to rush in, or perhaps one expects oneself to help.” - “I work within the healthcare, so for me it was just natural to come to the rescue immediately.” - “I was not sure whether I should start to compress the chest of the victim, and I felt I did not have enough skills.” - “We tried to confirm his pulses, but I was not sure enough, we spent about 2 minutes checking the victim’s pulse and breaths.” - “I worried about rib fracture of the victim.” - “No doubt, most of the people worried about it. I helped him [victim] today, but I did not perform well enough. If the victim had died after few hours or few days, is that my fault?” - “I was allowed to rescue him [victim], but I was not a professional. I was afraid of causing more problems.” - “Even if I had knowledge, talent, and skill of dealing with such events, I worried…… after I rescued you, I was accused and reparation was requested.” - “I sometimes chat with colleagues and family members about how doctors resuscitate when they talk about life and death, and sometimes I can see it occasionally on the Internet, and then I can almost know a little bit about it, but in the end, I know this knowledge is right or wrong, and there is no one to guide me.” - “When I was in school, I learned and became proficient in this knowledge of first aid, which is essential for us to engage in this profession, but I have not encountered it after work, so I have somewhat forgotten it, and some new ideas and knowledge are not up to date.” - “I usually like to watch TV, brush TikTok, etc, and occasionally play small advertisements on first aid knowledge, when I take the subway to and from work, I also see videos on the car that publicize first aid knowledge, but I can also remember some of them, not necessarily accurate, there is no kind of training professional and solid.” - “It’s been many years since I’ve been in the military and it is many years since I have had basic life support training, but apparently, it occurs automatically–this behavioral pattern.” - “I‘ve always been the type of person who just jumped into things and then we must take It from there. We should not be afraid of anything.” - “I have taken two basic life support courses, however a while ago, but the man who performed CPR didn’t do the things that I have learned, he held his hands next to each other and not on top of each other, and he just pressed a little in depth. I asked if I should take over, because he said that he never had tried it before. I hadn’t either, but I tried in on a course.” - “I stood at the window, because we were two people. And I did not really manage to call for help and I didn’t manage to. . . Actually, we have an AED here, but I didn’t. . ., It didn’t go through my mind at all (. . .). No, I didn’t even think about using it.” - “What I often think about is that when you do compressions, ribs get broken and I think that is so horrible. That’s really the thing that affects me the most, whenever I do compressions or when I think about cardiac arrest alarms.” - “It is a human being, whether he is an alcoholic, drug-addict, or businessman. In the end, there are no differences between us. Everybody has the same right to help. What if I had greasy hair and wore trainers and fell on the street, and somebody had judged me for being someone other than I am, and passed by because I did not look as though I was worthy of help? No. That was not in my thoughts. A human being is a human being to me.” - “I saw he was turning bluish, and that indicated that he wasn’t getting any air. So, that was the reason for starting CPR.” - “I believe we have a responsibility to our fellow human beings. I think so. We have, anyhow. So, we are a community which interacts. I believe that having a helping attitude makes us better humans.” - “I was a sports player before. I thought such experience caused me not to feel so nervous during the event.” - “I felt that I could do better than other people,……and I tried saving his life using the skills that I remembered.” - “When I was young, my family and friends affected my thought and behavior very much.” - One participant commented that his CPR certification had expired, preventing him – he thought – from coming to the aid of the victim. - Other participants were similarly concerned over liability and discipline (ie, in the workplace) if they attempted CPR if they had not been previously (or recently) trained. - “I wondered if I should get involved and was there a possibility that I could make the situation worse. I looked around to see if anyone was making a move to help him no one was. All this transpired in a matter of seconds.” - “No hesitation, I was trained to help.” - “It's my responsibility as a trained rescuer.” - “My job is to help in any way I can.” - “I realized that if I did not try to help him and he died, I would be responsible. His wife was looking quite frantic, and the victim was leaning back against the chair. I realized that the paramedics would never make it to him in time and I had to try and help him.” - “Death, with all its mystery in contemplation seems almost philosophical sometimes. Having your hands, lips, on what may well be a corpse is very real. But it's still a living organism and deserves our best effort. The Golden Rule applies here.” - “You would want somebody to help you if you were in that situation, so don't hesitate.” - The respondent described initially thinking that it would be beneficial to use some type of mouth shield and started looking around for possible barriers to use. A shirttail was tried but was unsatisfactory and so was abandoned in favor of giving the best effort to the person who needed lifesaving measures. This respondent was unable to indicate any specific disease that was a concern. - “As police officers, we also have to protect the safety of the people, when we see someone needs us, we will rush forward, time is life, and everyone has the right to live.” - “When I saw someone faint, I actually froze first, not knowing what was happening, but my years of education told me that I had to go save him and get him out of danger sooner.” - “My mother has more than ten years of coronary heart disease, during which she has committed several times, as his family can understand at this moment, I also know that early resuscitation will have a greater hope to live, seeing him I feel like seeing my mother, so I did not hesitate to go, I am doing my best to save, I hope he wakes up quickly.” - “When you learned it (CPR) in the military, many years ago, you practiced on a mannequin. And there, you don’t get the same impression of what actually will happen.” - “I was concerned with my handling of the situation.” - “I felt helpless to tum this guy around. I was just going through the motions.” |
| 2. Psychological tolerance during CPR | a. Psychological conflicts in complex situations | - “When the event occurred, my colleague was here, even if they did not do anything, they supported me, and I felt I did not need to take all the responsibilities.” - “They just stood aside without doing anything. They just stood and watched. No one asked if they could do something. They were selfish.” - “They assured us we had done a great job and to please continue until they were ready to take over.” - “They pushed me out of the way.” - “But, as the dispatcher said to me on the phone, when you are alone, concentrate on the chest compressions, and only that. So, I did. But of course, if I had not had her on the line, I don’t know; I suppose I would have started chest compressions, anyway. But I wouldn’t have known if it was right or not. I felt I had control, because I had a competent person on the phone.” - “It was certainly an enormous help to me. The calm voice that told me what to do. Of course, it was fantastic help in that situation. For one thing, you don’t feel so alone; and you have a voice that gives you instructions without increasing the intensity by even a small amount; and she hears the panic in me. Do you see? Of course she does. So, she was a fantastic help. All my respect goes to her.” - “Oh no, I had no doubt about that. I wasn’t the only one there. There were three of us.” - “Yes, I did. It was not an issue. We didn’t talk about it.” - “Time seems to pass so slowly, one waits and waits but nobody comes.” - “I thought that I was better equipped to meet death, since I’ve worked so much with people who have eventually died, but then there had been others who could help, here I had to think through everything myself.” - “I just cried out, ‘is there anybody who can help me!’ but no, they just passed by.” - “The paramedics should realize that you develop a relationship with the patient and that the administrator of CPR should be comforted, consoled whatever term you want, maybe confided in.” - “The only thing, when calling 112, you get a little frustrated or a little annoyed in the situation. I know it’s a stressful situation. But then they tell you: “in a moment you will be redirected to a nurse”. That is not what you want to hear, I’d say, because then your only need is to know that an ambulance is on its way. And that it will come quickly. What I’m saying is, when I called, I was told in a rough tone that I had to wait for a moment, which is not okay, I think.” - “It was very good actually. She told me how I should do, or if I what I was doing was right. It was very nice to have support, even though it was not right next to me, it was through the telephone. So I did not feel all alone. When I was in the situation I just thought: “what can I do?”. But then she said: “you have to perform CPR” and then I thought: “I will do it.”. Otherwise I don’t know how I would have reacted. It gave me a sense of an inner peace.” - “It was very nice that the physician calmly talked to the people involved, while the other staff was packing up the patient. then he told me that my performance was perfect and I had given him (the patient) a very good chance.” - “I just cried out, ‘‘is there anybody who can help me!’’ but no, they just passed by.” - “One person told me that I ought to compress the chest continuously. I had the courage to keep chest compressions.” |
|  | b. Desire to be assisted by others | - “When I heard a rib crack, then I wondered if I was doing it correctly, but still I went on. I hesitated for a second, I really did, and just then I wondered if I was doing more harm than good.” - “But there was the uncertainty of doing anything that would make things worse. That’s what I was worried about then.” - “The standard operating procedures had appeared in my mind……I felt calm……and then I completed it.” - “It was difficult to control my strength.” - “The skills of chest compression on the victim were the same [when compared with compression on a mannequin’s chest].” - “I knew I should perform CPR step by step, but I felt tempted to repeatedly check the patient’s consciousness and breathing in reality.” - “When I compressed the chest for the victim, the clothes were under his head, but it was not a serious interference and I ignored it when I rescued the victim.” - “I also almost remembered. I remember that I let my colleague call 120, I patted him and shouted at him, but he did not respond, I saw that he was not breathing, I let him lie flat, pressed him hard, with no professional training, also do not know the weight, I will learn first aid knowledge, which is too important.” - “I am the first time in an out-of-hospital encounter this situation, but we are medical professionals, in accordance with the operating steps step by step, chest cardiac compressions, and then open the airway, and finally mouth-to-mouth artificial respiration, I think the training is still useful, will be more calm step by step to operate, will not be particularly busy, helpless feeling, the possibility of success is also greatly improved.” - “In the rescue process, I still think more, we do not know this person, I do not know if he has any infectious diseases, if there is any impact on me after contact, what can I do, the state also does not provide that the disease caused by saving people will give the rescuer compensation, but even so I also cheer myself up in the heart, but it seems that the effect is not very good.” - “This person's family is watching from the side, I am not professional, or very nervous, afraid of aggravating the disease, family trouble, or getting into trouble with the lawsuit, the state does not have relevant laws to protect me, I will certainly bear part of the responsibility, I do not know how to adjust this concern, is to do a step to see a step.” - “To be honest, the pressure was quite high, afraid that they do not do well, counterproductive, my own family will be a little better, and for others, I am worried about finding myself in trouble. Saving their lives to my thousands of thanks, and if I don't bring him back to life, his family will let me compensate. The country did not stipulate that saving people could be exempted from the responsibility of causing all the consequences, the more I think the more nervous, the more afraid, and there is no one around me to cheer myself up, in the face of life, I know I should do my best, but I just can't adjust mentally.” - “I was trying to do it like in the class but my technique was falling apart. I felt like I wasn't helping.” - “Agitated but pleased that my training was coming back to me.” - “He took a big breath and we turned him side ways and I thought he was alive.” - “We actually interrupted CPR because we saw the breathing, but we shouldn’t have stopped, should we?” - “What if he’s alive and you’re doing CPR? And then you’re in big trouble because then you’re potentially killing him.” - “But I figured anywhere around the chest is a good thing.” - “We started doing compressions and [my coworker] is shouting at me ‘Are you doing compressions?’ and I said ‘Yes!’ and she said ‘Are you counting?’ and I said ‘Yes! Stop shouting at me! I’ve lost count!’” - One participant adopted a mantra to keep herself on pace when delivering chest compressions, repeating 3 words over and over: “Please. Don’t. Die.” - “When I looked at it, ‘Green Means Go’ and ‘Red Means Stop’ So I had to do a double take to make sure I didn’t turn off the machine. Because the buttons are so small I had to read ‘Power’ (the green button) and ‘Shock’ (the red button).” - “I was really guessing as to what the machine wanted me to do.” - Others tended to misinterpret the prompts from the defibrillator, thinking the beeping metronome meant that they were performing compressions incorrectly. - “We flipped him onto his right side and there were still fluids coming out and it was like a deep rasping noise and you could hear he was trying to breathe but he couldn’t. I have little kids that have choked, so I just pounded him on the back and when you did that, you’d hear him do this [large gasp] so there was air going in, which was good so we just kept doing that and as soon as that stopped a little bit, we just put him on his back and then I jumped on his chest and tried to do the compressions. We had to flip him again because the liquid started coming out of his mouth again so we were pounding him on his back again, so that happened three times.” - “So there we were in the middle of the motorway doing compressions, we were like, just right there, in the middle of the road, doing compressions.” - “He had already initiated CPR. But, you know, on and off, because she was gasping. It was as if she exhaled more than she inhaled. And I noticed that her mouth was frothing and her eyes were open, there was no pupillary move.” - “She became so very blue, so I thought, it’s not much use continuing, because I thought that she was dead, I had that feeling already then, that there was no longer any life, but still you go on.” - “I don’t know what it feels like to receive cardiopulmonary resuscitation…after all, I don’t know how bad his condition was, if it was any use saving him because, for my own part, if I become seriously ill, I don’t want to be saved. So I think, that perhaps I had saved him only to become a vegetable.” - “I didn’t feel like I was doing anything useful. I blew and blew, I didn’t expect that he would sit up and say hello, but that there would be some reaction, that he would start to move, but there was no life in him. I saw that his eyes were sort of half open and looked completely dead. They were completely cloudy and I thought it was a bit scary, because she looked so very awful, so I became a bit afraid at the same time. I found it unpleasant, because I thought that she became bluer and bluer and her eyes stared straight out and…No, I thought it was a bit scary and her mouth, it was a bit unpleasant to put your own mouth to hers, I really thought so.” - “No, I never hesitated, but I found it sickening if I may say so, when she started to throw up…but that was the only thing that was repugnant, so to speak.” - “I became nauseated very much. The victim’s vomits contained a little blood.” - “When we arrived at the hospital, I cleaned my mouth in the washroom because he was a stranger. Cleaning the mouth is a necessary behavior.” - “We just kind of looked because you couldn’t do mouth to mouth because there was stuff [emesis] coming out of his mouth, so I just sort of shoved my hand under his nose to see if there was anything. It was messy, I was not prepared for that.” - “Only momentary" hesitations until the problem could be removed: "For a brief second I wiped saliva off his mouth.” |
| 3. Perceived experience after CPR | a. Post-resuscitation perceptions | - “From one end to the other, this was a success story, although it did not go well.” - “I wondered about the outcome. I looked in the newspaper. I checked to see if the flag was flying at half-mast. Did we manage this, or did we not?” - “I would rather have known. Because when you eventually find out, you go through an emotional dealing with it, which earlier would probably have prevented some of the mind torment.” - “It doesn’t mean that I want to know the name of the patient. That doesn’t interest me, but if society wants me to help, they can’t cut me off with banal statements about the duty of confidentiality.” - “I think about it all the time. I check my mobile phone and wonder when they will call, but they never do.” - “But I didn’t want all this. Some flowers would have been fine. It was enough for me to know that he is alive today.” - “I was thinking and dreaming a lot about him. He was always in my head. Until the day he came with flowers and a card. Then I stopped thinking about him.” - “The victim survived. I feel happy. I will try again next time.” - “The outcome of the victim is good, I feel I am more confident to rescue other people.” - “Why didn’t it work?” - “When I heard word [that] he passed, that was hard, because I, and I found out that night, so I thought ‘Is it because of me?’ Is it because I didn’t run fast enough? Was I supposed to start the process?” - “Exultant! We got him breathing and got his heart beating by the time the paramedics arrived and the guy was cold and blue.” - “I succeeded, I feel that my learning was used in a practical way, I fulfilled my mission, I faced life in the future with more confidence and enthusiasm, and I found my value in life.” - “I do not know the man and it doesn’t matter to me, but for one’s own sake and self-perception and trying to understand whether you’ve done a good job and if your efforts have been successful, It would have been nice to know, but I have to do without.” - “What did I do wrong, since I couldn’t save him?” - “I did what I could but was afraid of doing the wrong thing, but still I dared to do what I did.” - “I’m glad that I dared, or that we dared, to intervene.” - “It was a power. I just felt that I had to do something.” - “It’s just the reaction, the human reaction one has that one just has to intervene.” - “A sense of powerlessness, that’s what one feels. One thought that one masters the situation and then one doesn’t. One is not oneself, one is somehow at a loss.” - “It was an incredible relief when the flowers arrived. It was proof that he was alive and was doing all right.” - “I want to help people. But I do not want to go to prison. They may even kill me later. They will simply say that I have killed him.” - “I sometimes think: “what do I have to remember–oh yes - I have to check if the airway is clear before I start CPR,” right. It has created an awareness, at my job, about how updated our first aid course is and should we plan to take a new one. And at home–maybe someone might also benefit from it.” - “I think we stayed there for almost an hour after the ambulance had left. She had some problems walking, this old lady. So we put lots of bottles of juice and stuff by the armchair where she used to sit so she wouldn’t have to walk about and fetch things, because that was what her husband had done for her, before he had his cardiac arrest.” - “You know what, it does not matter, it can happen. So all die at some point after all. So whether it‘s today or tomorrow not. Whether young or old.” - “Twenty years ago I could have lifted him out from under the hood. Another male appeared and we both dragged him out and laid him on the concrete.” - “I hope I'm not screwing this up.” - “I was confused about the resuscitation steps [After the event,] I asked the professional provider the questions, ‘what to do better under that circumstance?’ and [then I asked] another question, I understood the resuscitation skills.” - “I was confused about the resuscitation step [After the event,] I asked the professional provider the questions, ‘what to do better under that circumstance?’ and [then I asked] another question I understood the resuscitation skills.” - “I believe I can make a quicker decision to start CPR next time.” - “After the event, I believed that I took these resuscitation skills more seriously than other people.” - “I was very concerned about dying, to die just like that, so horrible and in public and because of my mistakes, that he died because I made mistakes. I was obsessed about this.” |
|  | b. Subsequent impact | - “I can’t go in the room [where the incident occurred], I can’t even look at that door because my anxiety goes through the roof.” - “I had given the police my statement and I got to my car and I cried and I called my father and I thought to myself ‘Am I allowed to share this? Am I allowed to tell anybody?’ and like ‘How am I supposed to keep this to myself’ and ‘Did I sign anything saying that?’ and I couldn’t drive home because I was shaking, so I sat in my car.” - “I’m still messed up. I don’t sleep. I can’t see the color blue, like certain blues just freak me out right now. Umm, like sometimes you’re having a conversation, you don’t feel like you’re even in the room. It’s weird, I can’t even explain it.” - “I felt terrible. Plus I was throwing up. I probably saw the victim's face in my dreams for a month.” - “And I just have to pass by the place, I don’t think there has ever been a time when I haven’t thought about it, I mean, every time I walk past or drive past this place, I think about this.” - “I did not want to work, it was a little terrible, I left the site soon.” - “When I shared the experience, I had a flashback of the scenes, it was terrible.” - “I remember, during work, for a long time, I was always by myself, for many weeks.” - “I didn’t sleep. I kept having nightmares, dreamt the same situation with different outcomes, and then, I went through the same dream with family and friends, and it was just tiring. I wasn’t sleeping at all.” - “I lost weight. I found it difficult to eat. Many things changed because of this.” - “The fact that it was night-time would have stayed in the back of my mind. Perhaps I wouldn’t have dared to stop, because of my own fear that somebody might not really need my help, but rather might want to attack me.” - “I want to help people. But I do not want to go to prison. They may even kill me later. They will simply say that I have killed him.” - “I can be disturbed by this ending, CPR is successful, I am very happy, they are also very happy but failed, the family has been crying there, my heart is not a taste, tears can't stop falling, work encountered similar situations, I will also involuntarily think of the previous scene, I will escape, do not want to touch again, the mood will be very low, can not do anything.” |
| 4. Enhancing psychological resilience | a. Coping strategies | - “We simply closed our department afterwards and talked about it and asked everyone to spend the night with friends or family so they didn’t have to spend the night alone. No matter how cool they were. Because then it doesn’t stay in your mind and body. I think we talked it through and ended it in a good way.” - “I spoke to my mother-in-law about it. I just told her what happened. I just had to calm down and so on. I was still shaken. She said: “it happens, it’ s old people, it can happen, after all. So I am a bit more calm now.” - “We basically spent the weekend partying and retelling the story and generally feeling like heroes.” - “The more people I share with the resuscitation experience, the less I feel the impact of the event.” - “It’s reality; it’s part of life.” - “I tried.” - “It’s like everything around you have just changed. It’s not just a workplace anymore, you kind of feel like you have a bigger responsibility here.” - “After hearing what happened, I finally felt at peace and I went home and I had a good night’s sleep for the first time in days and I just felt better. It’s a very sad situation, but I realize that I couldn’t have done anything different. I’ve got the answers. I’ve got the information I needed. I’ve had the rest, now it’s time to move forward.” - “I also think it was good timing, it might have happened the day before or an hour later, when he would be driving in the truck, right? So we’ve talked a lot about that. Luckily, it happened right there after all.” - “I’ve reflected a lot about if what I did was right or wrong and how I reacted and how to react another time. The debriefing enables me to be more confident in that what I do or have done is right.” - “It didn’t affect me so much when I gave her CPR, but after I spoke with you, I reflected on, if I had experienced something that can make me react better the next time, I’m in such a situation.” - “It made me feel secure about. It was really nice to know that I would be called back. Because I did not actually know how I would react to such things. So it was a kind of an insurance for myself, that if anything would happen, for example if unpleasant thoughts would come up, I knew that I would be called back (. . .) I knew that I would be helped to my feet again. That was really really nice.” - “That was very good, because it was a first time experience. I had never tried it before, so I was shocked that day - that she called back, because I had a bad conscience - what could I have done more and things like that. And I thought about whether what I had done was good enough, and having that conversation with the nurse afterwards that I had done well enough and I could not have done more. It was actually very nice to hear and be reassured about what I did was good enough.” - “I cannot speak to a friend the same way I spoke to the nurse. I don’t think so; I think it was nice to talk to a professional who knows what it is about 100 percent. It’s fine that you can sit down and talk to a friend but people who haven’t experienced it or know what it is about do not really understand it.” - “When you get to cope sooner, then you don’t carry the situation the same way. and may be more capable to react another time. Because you have been reassured that you did the right thing. Then you don’t fear that it was your fault, if the person died.” - “It’s about acknowledgement. It’s about motivation. It’s about being trained to save lives, and when we do, nobody cares.” - “It was a kind of first aid to me and I liked that. Because when standing in the middle of it all and experiencing all that, the ambulance crew have to help other people. But no one helped me.” - “I spoke a lot about this incident to everybody I met. That was my way of doing it.” - “I told my family about the whole resuscitation course, they said they were proud of me.” |
|  | b. Previous experience performing CPR | - “The first aid courses, they are sometimes a little superficial, I think, and the courses don’t prepare people for the trauma of seeing injuries and the fear and panicin people’s eyes when it happens. It’s a special situation for which preparation could be incorporated in the first aid courses, so people don’t freeze as much. There is this anxiety of doing anything when experiencing it for the first time.” - “A sterile mask be made available for mouth-to-mouth breath­ in g, one that is provided in the cost of the basic life support class and that could be kept by the students.” - “Stressing the spiritual and philosophical nature of death and the importance of speaking with someone after the experience.” - “For me, the whole incident opened up a Pandora's box as to the nature of life and death. The victim lived but it took me two years to realize that he lived instead of almost died. Somehow be able to stress a spiritual component.” - “More frequent classes,” more “affordable” classes.” - “Iower student to teacher ratio, lots of practice time and feedback opportunities.” - “Restaurants should have employees trained in both CPR and the Heimlich [maneuver] and provide a notice to the public that they do.” - “The need for an experienced provider to emphasize the true nature or "real-life aspects " of CPR, such as awkward position, difficult access to the person being resuscitated, secretions, and disfigurement.” - “If you are trained, but cannot use your skills, then the training is meaningless. You could consider preparing people on how surrealistic a situation it is, for example by showing videos with people who have experienced it, telling about the feelings related to it etc.” - “You have tried many things on the courses and things like that, it is - “Completely different. There you don’t think much about it, then you think “well okay, that’s so easy.” But to try it in real life–that’s entirely different. So I think it was really good to talk about, through the conversation, so how was it in real life.” - “This is different than a dummy (Resusci-Annie).” - “We all got the training, but this is (sighs) – until the occasion arises where you have to use it, you never know.” - “Because of the special nature of the work, our unit will regularly organize some training in first aid skills, but we are pressed on the model and have not actually operated, really encountered this situation, think the usual learning or too little, a little handy.” - “Trainers realistically cover the possible settings in which resuscitative attempts may occur.” - “We have a lot of things going on (on the workplace) regarding basic life support and, if we ought to have an AED, because we don’t have one. We have a lot of thoughts and ideas about what we should and shouldn’t do in the future. And we pressure our manager a little, because we think that we need a basic life support course.” |
